# Efficacy and safety of pyronaridine-artesunate (Pyramax^®^) for the treatment of uncomplicated *Plasmodium vivax* malaria in Northwest Ethiopia

**DOI:** 10.1186/s12936-022-04422-0

**Published:** 2022-12-31

**Authors:** Hussein Mohammed, Heven Sime, Henok Hailgiorgis, Melkie Chernet, Mihreteab Alebachew, Hiwot Solomon, Gudissa Assefa, Mebrahtom Haile, Samuel Girma, Worku Bekele, Geremew Tasew, Bokretsion Gidey, Robert J. Commons, Ashenafi Assefa

**Affiliations:** 1 Bacterial, Parasitic and Zoonotic Diseases Research Directorate, Ethiopian Public Health Institute, Addis Ababa, Ethiopia; 2 Department of Medical Laboratory Sciences, College of Medicine and Health Sciences, Wollo University, Dessie, Ethiopia; 3 National Malaria Elimination Program, Ministry of Health, Addis Ababa, Ethiopia; 4 USAID, Addis Ababa, Ethiopia; 5 World Health Organization, Addis Ababa, Ethiopia; 6 Global Health Division, Menzies School of Health Research, Charles Darwin University, Darwin, Australia; 7 General and Subspecialty Medicine, Grampians Health, Ballarat, Australia; 8 Institute for Global Health and Infectious Diseases, University of North Carolina, Chapel Hill, USA

**Keywords:** Pyronaridine-artesunate, *Plasmodium vivax*, Efficacy, Ethiopia

## Abstract

**Background:**

Declining efficacy of chloroquine for the treatment *Plasmodium vivax* malaria has been reported in different endemic settings in Ethiopia. This highlights the need to assess alternative options for *P. vivax* treatment with artemisinin-based combination therapy, such as pyronaridine-artesunate. This treatment regimen has shown high efficacy for uncomplicated malaria in both Africa and Asia. However, limited data are available from Ethiopia. This study was conducted to assess the efficacy and safety of pyronaridine-artesunate for the treatment of uncomplicated *P. vivax* malaria in Northwest Ethiopia.

**Methods:**

A single arm prospective efficacy study was conducted in the Hamusite area, Northwest Ethiopia. Fifty-one febrile adult patients with uncomplicated *P. vivax* malaria were enrolled between March and July 2021. Patients were treated with pyronaridine-artesunate once daily for three days. Clinical and parasitological parameters were monitored over a 42-day follow-up period using the standard World Health Organization protocol for therapeutic efficacy studies.

**Results:**

A total of 4372 febrile patients were screened with 51 patients enrolled and 49 completing the 42-day follow-up period. The PCR-uncorrected adequate clinical and parasitological response (ACPR) was 95.9% (47/49; 95% CI 84.9–99.0) on day 42. Two patients had recurrences [4.0% (2/49); 95% CI 0.7–12.1] on days 35 and 42. The parasite clearance rate was rapid with fast resolution of clinical symptoms; 100% of participants had cleared parasitaemia on day 1 and fever on day 2. All 16 (31.4%) patients with gametocyte carriage on day 0 had cleared by day 1. There were no serious adverse events.

**Conclusion:**

In this small study**,** pyronaridine-artesunate was efficacious and well-tolerated for the treatment of uncomplicated *P. vivax* malaria. In adults in the study setting, it would be a suitable alternative option for case management.

## Background

Despite malaria morbidity and mortality having reduced substantially in Ethiopia over the last decade, malaria remains a major public health problem [1]. Approximately 70% of the landmass has malaria with 52 percent of the population at risk of infection [2]. Transmission is highly seasonal and varied across the country. *Plasmodium falciparum* and *Plasmodium vivax* are co-endemic in Ethiopia, with proportions of 60% and 40%, respectively [3]. These proportions vary between places and seasons. The estimated prevalence rate of *P. vivax* is 7.9% by systematic reviews with a wide distribution in the central west region extending to the Northwest and Southwest regions of Ethiopia [4].

According to the current national malaria diagnosis and treatment guidelines for Ethiopia updated in 2020, the first-line drug for treatment for uncomplicated *P*. *vivax* malaria is chloroquine (CQ) complemented by primaquine (PQ) (0.25 mg/kg/day) for 14 days for radical cure [5]. Artemether-lumefantrine (AL) is the first-line drug for treatment for uncomplicated P*. falciparum* malaria, followed by single-dose PQ (0.75 mg/kg) for gametocyte clearance, and the recommended regimen for mixed infection of *P. falciparum* and *P. vivax* is AL co-administered with PQ for 14 days. Dihydroartemisinin-piperaquine is recommended as second-line treatment for both *P. falciparum* and *P. vivax* malaria.

The first report of chloroquine resistance in *P. vivax* was in 1989 from Papua New Guinea [6]. Evidence of resistance has continued to spread across multiple regions [7] and has reached a concerning prevalence in some locations, requiring changes to first line treatment [8–10]. However, CQ resistant *P. vivax* has been less prevalent in Africa with the first report in Ethiopia in 1996 [11]. Recent studies have reported declining efficacy of CQ for the treatment of *P. vivax* from different endemic areas of Ethiopia, with the risk of recurrence at day 28 ranging from 7.5% to 22% [12–14]. The rising occurrence of treatment failures highlights the importance of investigating alternative anti-malarial options, such as the oral artemisinin-based combination pyronaridine–artesunate [15].

Several studies have investigated the safety and efficacy of pyronaridine-artesunate treatment for *P. vivax* in Africa and Asia [16–20]. However, there are no published data available from Ethiopia. This is the first efficacy study of pyronaridine-artesunate study reported for the treatment of uncomplicated *P. vivax* malaria in Ethiopia.

## Methods

### Study area and period

**Fig. 1:**
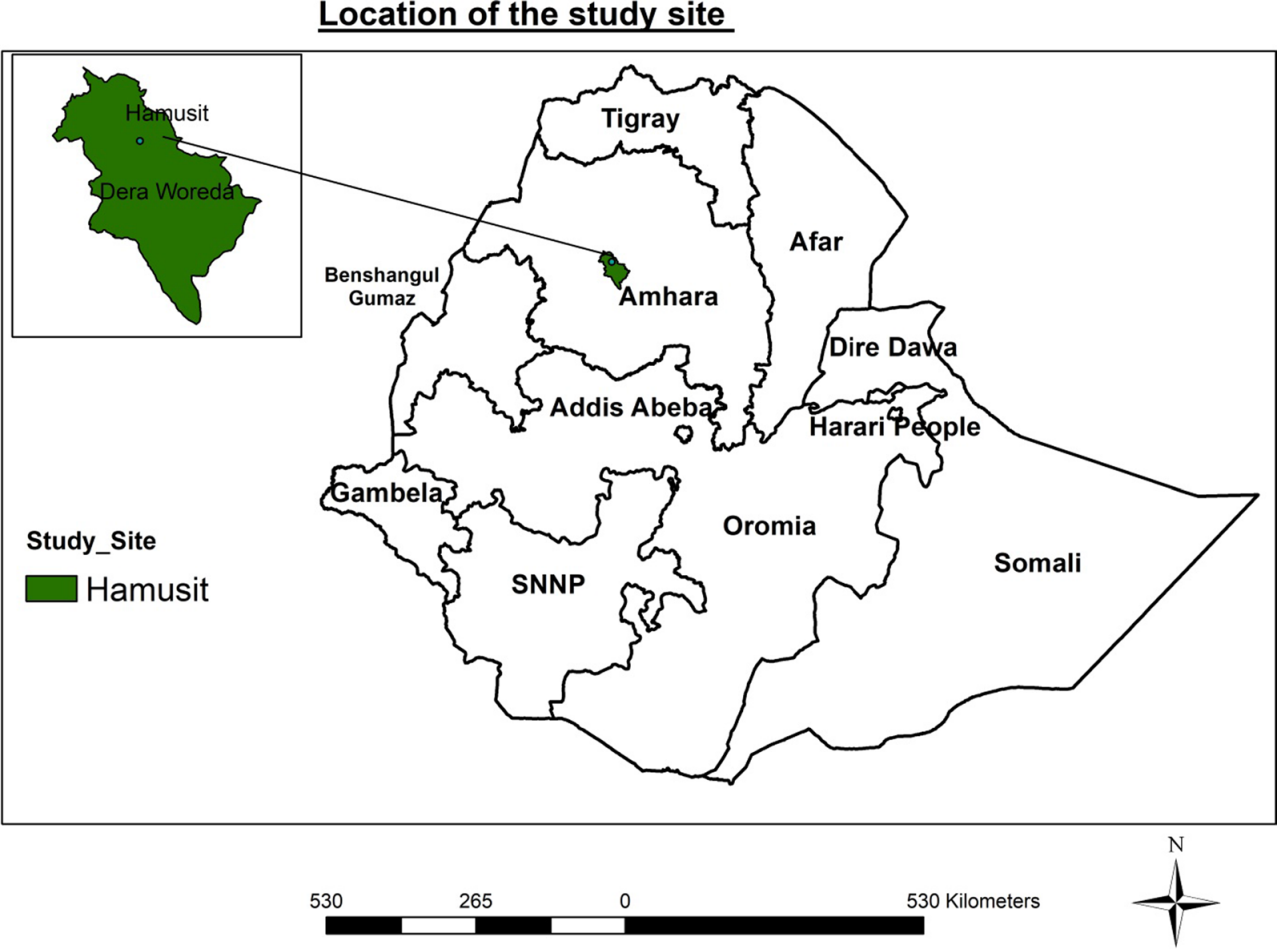
Map of study site in Northwest Ethiopia (Green colour shows study area)

This study was conducted between March and July 2021 at the Hamusit Health Centre, Dera Woreda, South Gonder in Northwest Ethiopia (Fig. 1). The study area is located about 38 km from Bahir Dar town and 590 km from Addis Ababa. It is located at 11° 43′ 0ʺ North and 37° 38′ 0ʺ East. The catchment population of the study area is about 54,940 people. The altitude of this area is 2077 m above sea level and transmission is highly seasonal with marked instability. The area has a mean annual rainfall of 1300 mm and mean annual temperature 26 °C (South Goner Health Office report). In 2017, there were 387,096 cases of malaria reported in the Amhara region, and 167,079 cases reported in the North Gonder zone [21].

### Study design

This study was a single arm prospective therapeutic efficacy study based on standard World Health Organization (WHO) therapeutic efficacy protocols [22], to evaluate the clinical and parasitological responses of uncomplicated *P. vivax* malaria to pyronaridine-artesunate. Patients who met the study inclusion criteria provided informed consent, were enrolled and were then treated at the health centre with pyronaridine-artesunate and monitored for 42 days.

### Inclusion criteria

Inclusion criteria were: age ≥ 18 years, monoinfection with *P. vivax* detected by microscopy, parasitaemia ≥ 250 asexual parasites per microlitre of blood, axillary temperature ≥ 37.5 °C or history of fever in the past 24 h and permanently living within the health centre catchment area (5–10 km radius) during the study period.

### Exclusion criteria

Exclusion criteria were: mixed infection (both *P. falciparum* and *P. vivax*), evidence of severe malaria, clinical signs and symptoms of hepatic injury (nausea and abdominal pain associated with jaundice), and known severe liver disease (cirrhosis), known allergy to the study medication, evidence of a non-malaria febrile illness (otitis media, tonsillitis, measles, measles, acute lower respiratory tract infection, severe diarrhoea with dehydration), took an investigation anti-malarial drug within 28 days, haemoglobin concentration < 8.0 g/dL, known pregnancy and breastfeeding.

### Sample size determination

The required sample size was determined according to the WHO protocol [22]; the sample size calculation assumed a 95% cure rate of pyronaridine-artesunate on day 42. With the desired precision of 5% and a 95% confidence interval (CI), an initial sample size of 73 was calculated. Assuming an additional 20% for loss to follow-up and withdrawal, at least 88 patients were planned to be recruited.

### Treatment and follow-up

Patients with *P. vivax* malaria, who fulfilled the inclusion criteria were treated with a 3-day course of daily pyronaridine-artesunate (Pyramax^®^; Shin Poong Pharmaceuticals Co, Republic of Korea) provided as a tablet (180 mg pyronaridine and 60 mg artesunate). Patients were dosed according to body weight: 20 to < 24 kg, one tablet; 24 to < 45 kg, two tablets; 45 to < 65 kg, three tablets; ≥ 65 kg, four tablets per day. Treatments were given under direct supervision and patients were observed for one hour after each dose to monitor for vomiting or other side effects. Vomiting within 30 min after administration, led to re-administration of the full dose and vomiting between 30 min and one hour led to re-administration of a half dose. Patients who vomited a second time were withdrawn from the study and received parenteral artesunate therapy administered according to national guidelines [5].

Patients were followed-up daily for the first 3 days after the first dose (day 0) and then weekly on days 7, 14, 21, 28, 35 and 42. Patients were also assessed on any unscheduled visit if symptoms occurred. Adverse events and severe adverse events were defined according to the WHO protocol for monitoring the therapeutic efficacy of anti-malarial drugs [22]. PQ treatment was initiated for patients at the end of their follow-up.

### Laboratory procedures

Finger prick thick and thin blood smears were taken from all participants and prepared on two slides for detection of parasites at all follow-up visits. The first slides were prepared by staining with 10% Giemsa for 10–15 min for initial screening. The second slides were stained using 3% Giemsa for 30 min and read by two independent laboratory technicians from the health centre. If results were discordant, a third reading was performed by a senior laboratory technician. Parasite densities were recorded for all positive slides. The number of asexual parasites was counted per 200 white blood cells (WBC) and parasitaemia was estimated assuming WBC counts of 8000/µL. Gametocytes were counted against 500 WBCs. Before any blood smear was interpreted as negative, two hundred oil-immersion high-power fields on the thick film were read [23]. To ensure microscopy quality, all slides were cross-checked by a WHO-accredited microscopist at the Adama Malaria Control and Monitoring Centre. Haemoglobin concentrations were measured using a portable spectrophotometer (HemoCue^®^, Angelholm, Sweden) on days 0, 14, 28, and 42. Females aged 12 years and older were screened for pregnancy before enrolment.

### Treatment outcomes

Efficacy outcomes were based on an assessment of the parasitological and clinical outcomes of anti-malarial treatment according to the WHO guidelines [22]. Patients were classified as having early treatment failure, late clinical failure, late parasitological failure, or ACPR, defined as the absence of parasites without previous treatment failure. The ACPR was determined on day 42 for *P. vivax*. Safety outcomes were the proportion of any symptom occurring during the study period.

### Statistical analysis

Data were double-entered into the WHO Excel spreadsheet designed for therapeutic efficacy data. IBM SPSS (version 24) software was used to generate descriptive statistics (mean, standard deviations, and percentages). Based on WHO guidelines the ACPR on day 42 was calculated using the Kaplan–Meier method [22].

### Ethical approval

The study was approved by the Institutional Review Board (IRB) of the Ethiopian Public Health Institute (EPHI). Written informed consent was obtained from all of the study participants; signed by adults after understanding in their local language. Participant identities were kept confidential throughout.

## Results

### Baseline characteristics

**Fig. 2:**
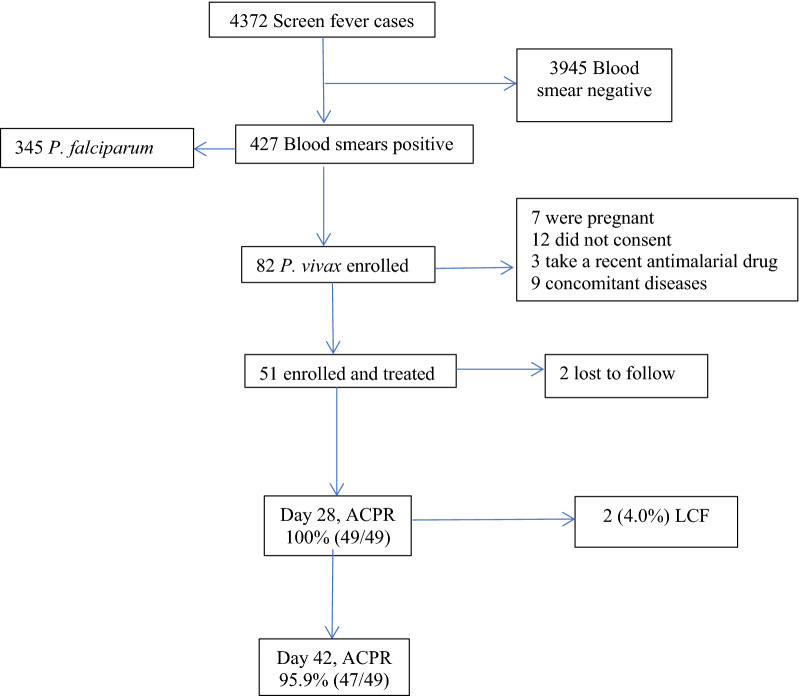
Study profile. Screening, Enrollment, and Follow-up of Patients

A total of 4,372 febrile patients were screened at Hamusit Health Centre between March and July 2021 (Fig. 2). Of these 427 (9.7%) were malaria slide positive; 345 (81%) *P. falciparum* and 82 (19%) *P. vivax*. Out of 82 *P. vivax* infected patients, 31 (37.8%) were excluded (7 were pregnant, 12 did not consent, 3 take a recent anti-malarial drug and 9 had a concomitant disease). Thus, 51 patients were enrolled and treated with pyronaridine-artesunate. There were 2 (3.9%) patients censored during the follow-up period due to loss to follow-up. The target sample of 88 patients was unable to be achieved due to the study being conducted after the peak transmission season.

The baseline characteristics of the study patients are presented in Table 1. The mean age of patients was 29.1 years, with 68.6% (35/51) male. The geometric mean parasite density was 3891/μL, and 31.4% (16) of participants had baseline gametocytes detectable by microscopy. All participants were febrile with a mean baseline body temperature of 38.3 °C (standard deviation (SD) 1.01). The mean haemoglobin concentration at baseline was 13.1 g/dL (SD 1.93) and on day 42 was 13.2 g/dL (SD 1.65). There was no significant mean haemoglobin difference between baseline and day 42 (mean difference = 0.08; *p* = 0.515). 76.5% (39/51) of patients reported a previous episode of malaria.

**Table 1:** Demographic and clinical characteristics of the study patients in Northwest, Ethiopia

Characteristic	Value
Number of patients	51
Mean age, year (SD) [range]	29.1 (± 11.1) [18–65]
Male sex, n (%)	35 (68.6%)
Mean body temperature, ^o^C (SD) [range]	38.3 (± 1.01) [37.3–41.4]
Mean bodyweight, kg (SD) [range]	52.4 (± 5.56) [45–67]
Mean hemoglobin levels, g/dL (SD) [range]	13.1 (± 1.91) [8.6–16.7]
Geometric mean asexual parasitaemia, per µL [range]	3891 [256–56,889]
Presence of gametocytes, n (%)	16 (31.4%)
Previous malaria attack, n (%)	
Yes	39 (76.5%)
No	12 (23.5%)

*SD* standard deviation, *dL* deciliter, *µL* microlitre

### Efficacy

#### Primary outcome

The overall day 42 PCR-uncorrected ACPR was 95.9% (47/49; 95% CI 84.9–99.0). Two patients were treatment failures (4.0%, 2/49/49; 95% CI 0.7–12.1) with late clinical failure on day 35 and day 42 (Table 2).

**Table 2:** Per-protocol analysis results of PCR uncorrected pyronaridine–artesunate efficacy against *P. vivax* in Northwest Ethiopia

Treatment outcome	Value
Early treatment failure, n (%)	0 (0)
Late clinical failure (N = 49), n (%)	2 (4)
Late parasitological failure, n (%)	0 (0)
Adequate clinical and parasitological response (N = 49), n (cumulative risk %^a^; 95% CI)	47 (95.9; 84.9–99.0)
Lost/withdrawn, n (%)	2 (3.9)
Total per protocol, n (%)	49 (96.1)

*CI* confidence interval, *n* number of study participants

^a^Calculated by Kaplan–Meier method

#### Secondary outcomes

**Fig. 3:**
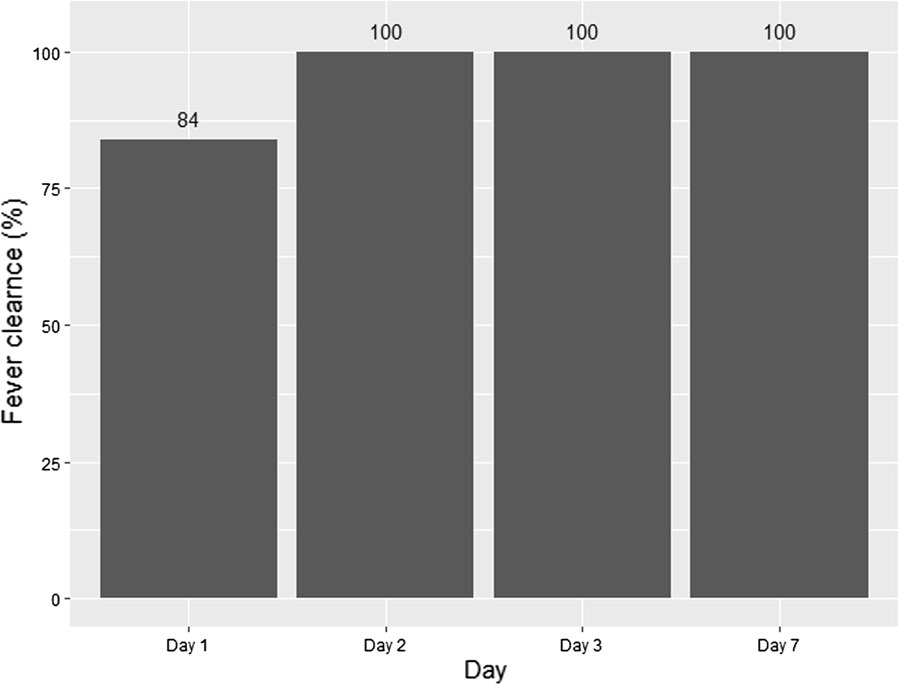
Fever clearance rate during efficacy study of pyronaridine–artesunate for the treatment of P. vivax in North West Ethiopia, 2021

We assessed fever and parasite clearance time for the first seven days to detect any delayed treatment response. All patients had cleared their parasitaemia and gametocytaemia by day 1 (within 24 h). Fever had cleared in all patients by day 2 (Fig. 3).

### Presence of adverse events

No severe adverse events were recorded following the administration of pyronaridine-artesunate. Some patients had symptoms on day 0 consistent with clinical signs of malaria, including headache 31.4% (16/51), cough 5.9% (3/51), anorexia 3.9% (2/51), vomiting 3.9% (2/51), abdominal pain 3.7% (7/51), nausea 2.0% (1/51), diarrhoea 2.0% (1/51) (Table 3). These clinical symptoms rapidly declined at day 3, with a small number of the patients still having headache 4.0% (2/50) and cough 4.0% (2/50). By day 7, clinical symptoms had resolved in all but one patient who developed cough after commencement of pyronaridine-artesunate, although its relatedness to pyronaridine-artesunate was unclear.

**Table 3:** Presence of symptoms during pyronaridine–artesunate efficacy study for *P. vivax* in Northwest Ethiopia, 2021

Symptom	Frequency				
	Day 0 n (%)	Day 1 n (%)	Day 2 n (%)	Day 3 n (%)	Day 7 n (%)
Headache	16 (31.4)	8 (15.7)	4 (7.8)	2 (4.0)	0 (0.0)
Cough	3 (5.9)	3 (5.9)	2 (3.9)	2 (4.0)	1 (2.0)
Abdominal pain	7 (13.7)	3 (5.9)	0 (0.0)	0 (0.0)	0 (0.0)
Anorexia	2 (3.9)	2 (3.9)	0 (0.0)	0 (0.0)	0 (0.0)
Vomiting	2 (3.9)	0 (0.0)	0 (0.0)	0 (0.0)	0 (0.0)
Nausea	1 (2.0)	0 (0.0)	0 (0.0)	0 (0.0)	0 (0.0)
Diarrhoea	1 (2.0)	0 (0.0)	0 (0.0)	0 (0.0)	0 (0.0)
Total patients assessed	51	51	51	50	50

## Discussion

This study found pyronaridine-artesunate to be highly efficacious for the treatment of uncomplicated *P. vivax* malaria in Northwest Ethiopia. All patients treated with pyronaridine-artesunate were parasite-free within 24 h, with 2 (4%) of the 49 patients who completed 42 days follow-up having a *P. vivax* recurrence.

Recurrent parasitaemia can be caused by recrudescences from the same isolate, reinfection from a new infection, or relapses from hypnozoites in the liver [24]. Similar to most *P. vivax* therapeutic efficacy studies, this study was undertaken in a malaria-endemic setting, with patients at risk of new infections during the follow up period. This risk is related to the duration of follow up, host immunity, and level of transmission [25].

In general, *P. vivax* malaria is more difficult to control and eliminate than *P. falciparum* because it can relapse from dormant liver stages after clearance of the initial infection. Effective cure of vivax malaria requires treatment of both its schizontocidal and hypnozoiticidal stages*.* Pyronaridine-artesunate has schizonticidal efficacy but lacks activity against the *P. vivax* liver stage hypnozoites. To effectively prevent relapses and minimize the risk of local transmission, pyronaridine-artesunate needs to be administered with a hypnozoiticidal agent, such as primaquine.

The present study had two late clinical failures recorded after pyronaridine-artesunate treatment at day 35 and day 42. Genotyping of polymorphic loci is undertaken routinely in *P. falciparum* drug efficacy studies to distinguish new infections from true parasite recrudescences [26]. However, for *P. vivax*, molecular typing recurrences cannot reliably differentiate between recrudescences, relapses or re-infections. This is because patients can harbour different populations of hypnozoites derived from repeated inoculations [27]. The difficulties in using genomic testing to differentiate recrudescences, relapses and new infections make it difficult to definitively identify the cause of the recurrences in the two patients in this study, however, their presentation on days 35 and 42 makes recrudescence less likely.

The speed of parasite clearance after anti-malarial treatment can be used to assess the therapeutic response to an anti-malarial drug [28]. Presence of parasitaemia on day 3 after treatment commencement is a key indicator of the possible emergence of artemisinin resistance [29]. In this study, all patients cleared their parasitaemia by day 1 after pyronaridine-artesunate suggesting parasites were highly sensitive to this agent. Furthermore, gametocytaemia was also cleared in all patients by day 1, suggesting pyronaridine-artesunate would be effective in preventing transmission to mosquitoes, thus enhancing control and elimination efforts.

Pyronaridine–artesunate have been associated with asymptomatic mild-to-moderate transient rises in liver transaminases in some malaria patients [30, 31]. Although hepatic enzyme levels were not examined in the present study, a recent study showed no increase in the risk of hepatic transaminase elevation with repeated pyronaridine-artesunate treatment and no manifestations of clinical liver disease [32, 33]. Similar to other recent studies, the patients in the present study did not demonstrate any clinical evidence of hepatic injury after treatment with pyronaridine–artesunate [20, 34].

There were some limitations to the current study. The first limitation was that pyronaridine-artesunate plasma concentrations were not measured, so the possibility that the recurrent parasitaemias might have resulted from insufficient drug exposure or parasite resistance cannot be determined. In addition, despite the study being conducted soon after peak transmission season, the planned number of patients could not be enrolled. This was further complicated by a low overall number of *P. vivax* malaria cases in the study area, which reduces the precision of the final results. Study participants were 18 years and older as the Ethiopia Food and Drug Authority didn’t allow the study to be conducted children younger than 18 years of age. There is the potential for variable efficacy with pyronaridine-artesunate in children which limits the generalizability of the study’s findings across all age groups.

## Conclusion

In summary, this is the first study to report the efficacy of pyronaridine-artesunate for uncomplicated *P. vivax* in Ethiopia. Despite a low number of cases, this study suggests that pyronaridine-artesunate is likely efficacious for the treatment of *P. vivax*-infected adults in Northwest Ethiopia, with a high parasite clearance rate and rapid clinical response. Following further studies, pyronaridine-artesunate may be considered as a potential anti-malarial as part of the national malaria control and elimination programme.

## Data Availability

The data analysed in this study are available from the corresponding author.

## Abbreviations

ACPR: Adequate clinical and parasitological response
ACT: Artemisinin-based combination therapy
NMCP: National Malaria Control Programme
WHO: World Health Organization
